# Early Regulation of Profibrotic Genes in Primary Human Cardiac Myocytes by *Trypanosoma cruzi*

**DOI:** 10.1371/journal.pntd.0003747

**Published:** 2016-01-15

**Authors:** Aniekanabassi N. Udoko, Candice A. Johnson, Andrey Dykan, Girish Rachakonda, Fernando Villalta, Sammed N. Mandape, Maria F. Lima, Siddharth Pratap, Pius N. Nde

**Affiliations:** 1 Department of Microbiology and Immunology, Meharry Medical College, Nashville, Tennessee, United States of America; 2 Food and Drug Administration, Silver Spring, Maryland, United States of America; 3 School of Graduate Studies and Research, Bioinformatics and Molecular Biology Core, Meharry Medical College, Nashville, Tennessee, United States of America; European Bioinformatics Institute, UNITED KINGDOM

## Abstract

The molecular mechanisms of *Trypanosoma cruzi* induced cardiac fibrosis remains to be elucidated. Primary human cardiomyoctes (PHCM) exposed to invasive *T*. *cruzi* trypomastigotes were used for transcriptome profiling and downstream bioinformatic analysis to determine fibrotic-associated genes regulated early during infection process (0 to 120 minutes). The identification of early molecular host responses to *T*. *cruzi* infection can be exploited to delineate important molecular signatures that can be used for the classification of Chagasic patients at risk of developing heart disease. Our results show distinct gene network architecture with multiple gene networks modulated by the parasite with an incline towards progression to a fibrogenic phenotype. Early during infection, *T*. *cruzi* significantly upregulated transcription factors including activator protein 1 (AP1) transcription factor network components (including FOSB, FOS and JUNB), early growth response proteins 1 and 3 (EGR1, EGR3), and cytokines/chemokines (IL5, IL6, IL13, CCL11), which have all been implicated in the onset of fibrosis. The changes in our selected genes of interest did not all start at the same time point. The transcriptome microarray data, validated by quantitative Real-Time PCR, was also confirmed by immunoblotting and customized Enzyme Linked Immunosorbent Assays (ELISA) array showing significant increases in the protein expression levels of fibrogenic EGR1, SNAI1 and IL 6. Furthermore, phosphorylated SMAD2/3 which induces a fibrogenic phenotype is also upregulated accompanied by an increased nuclear translocation of JunB. Pathway analysis of the validated genes and phospho-proteins regulated by the parasite provides the very early fibrotic interactome operating when *T*. *cruzi* comes in contact with PHCM. The interactome architecture shows that the parasite induces both TGF-β dependent and independent fibrotic pathways, providing an early molecular foundation for Chagasic cardiomyopathy. Examining the very early molecular events of *T*. *cruzi* cellular infection may provide disease biomarkers which will aid clinicians in patient assessment and identification of patient subpopulation at risk of developing Chagasic cardiomyopathy.

## Author Summary

About 30% of *Trypanosoma cruzi* infected individuals will develop Chagas heart disease cardiomyopathy which is similar to other cardiomyopathies. *T*. *cruzi* induced heart disease can lead to cardiac arrhythmias, heart failure and death. These cardiac disorders can be caused by several host-parasite interaction factors that lead to myocardial inflammation which remains poorly understood from the initial phase of infection. In this study, we challenged primary human cardiomyocyte (PHCM) with *T*. *cruzi* trypomastigotes and evaluated changes in gene and protein expression profile occurring early during the process of cellular infection. Paying attention to genes that have the potential of tilting the PHCM towards a fibrogenic phenotype, we observed that the parasite upregulates the expression of several transcription factors and cytokines/chemokines that have been suggested to be implicated in the development of fibrogenic responses. The validated microarray data were confirmed at the protein level where we demonstrated an increase in the protein expression of EGR1, SNAI1, JunB and IL6. Pathway analyses of our results show that early during *T*. *cruzi* infection, the parasite induces multiple factors that are profibrotic in PHCM. The operating fibrotic interactome presented in our work advances our understanding of the initiation of cardiovascular pathology in Chagas disease patients.

## Introduction

*T*. *cruzi* is an intracellular hemoflagellate protozoan parasite that causes Chagas heart disease. Chagas disease is endemic in Mexico, Central and South America where as many as 8 million people are infected [1]. It is estimated that 12,000 deaths result annually from Chagas heart disease and another 100 million persons are at risk of infection, mostly in Latin America [2]. Although endemic to Central and South America, Chagas heart disease is now considered to be a new emerging global health problem due to modern globalization and migration [3–7]. Autochthonous *T*. *cruzi* transmissions have now been reported in the US in both inland states and those sharing a border with Mexico, aiding in the dissemination of the disease out of its endemic region [8,9]. Some of the most devastating manifestations of the disease include acute myocarditis and chronic chagasic cardiomyopathy (CCC) which affects about 30% of Chagas disease patients. Chagas heart disease which is suggested to be caused by a prolonged, intimate host-parasite interaction leads to a spectrum of clinical manifestations including myocarditis, myocardial hypertrophy, vasculitis and fibrosis leading to heart failure [10,11].

Recent attempts to elucidate the molecular mechanism of *T*. *cruzi*-induced cardiomyopathy have largely employed *ex vivo* and *in vitro* murine cardiomyocyte models [12–14]. Furthermore, a three dimensional murine cardiomyocyte culture model was employed to show that *T*. *cruzi* infection increases both cellular and extracellular matrix components in cardiac spheroids mimicking fibrotic cardiac remodeling [15] reported in Chagas disease. Others used cardiac tissue from CCC and dilated cardiomyopathy patients in a gene expression profiling assay to suggest that chemokine signaling and interferon gamma (IFN-γ) in human cardiomyocyte could upregulate genes involved in Chagasic hypertrophy [16]. A common feature in patients with advanced cardiac failure is cardiac fibrosis and regardless of the etiology of the fibrosis, the early molecular events remain unclear.

Invasion of host cells by *T*. *cruzi* trypomastigotes was shown to be independent of TGFβ-induced gene expression but requires a fully functional TGFβ signaling pathway [17,18]. This theory is further evidenced by the fact that *T*. *cruzi* is able to directly activate latent TGFβ in order to infect cells [19] and this infection could be inhibited by a TGFβ inhibitor using a mouse cardiomyocyte model [20]. TGF-β1, a profibrotic cytokine, is a highly pleiotropic molecule that can regulate cell proliferation, migration, differentiation and apoptosis in a cell type and context-dependent manner [21]. The activation of this cytokine by the parasite during the infection process is very likely to be one of the mechanisms by which the parasite induces cardiac fibrosis. Cardiac fibrosis is thought to follow a similar pathway as fibrosis in other epithelial organs such as the liver, kidney, lung, solid tumors and the skin [22]; specifically through endothelial-to-mesenchymal transition (EMT) [23]. At the molecular level, TGF-β signaling promotes EMT [24] as a result of phospho Smad2/3 (pSmad2/3) import into the nucleus in association with Smad4 [25] to drive expression of profibrotic genes [26]. Host-parasite interaction in Chagas disease patients leads to a high level of circulating TGFβ (about 200ng/ml), which in addition to detection of pSmad2 in cardiomyocyte nuclei from Chagas disease patients implicates TGFβ in Chagasic cardiomyopathy [27]. The Early Growth Response (EGR) transcriptional regulator gene family (EGR1, 2, 3 and 4) may also be implicated in *T*. *cruzi* induced cardiac fibrosis and vasculopathy because they have been shown to regulate a wide spectrum of biological processes including muscle development and endothelial cell growth/migration. The EGR gene family has a high degree of homology at their conserved zinc finger DNA-binding domain which recognizes the EGR response element in the gene promoter of multiple target genes [28]. EGR protein expression can be induced in several cell types in response to injury and stress and has been shown to potentially play a role in fibrosis [29]. EGR1 and EGR3 were recently shown to be key mediators of fibrogenic responses acting through a TGFβ dependent non-SMAD pathway [30,31]; however, it remains to be elucidated whether this is a contributing factor in *T*. *cruzi* induced cardiac fibrosis. Others have suggested that the activation of extracellular-signal-regulated kinase (ERK) and AP-1 pathways in the hearts of *T*. *cruzi* infected mice could be implicated in cardiac remodeling observed in Chagasic cardiomyopathy [32].

In order to decipher the molecular mechanism(s) of interaction between *T*. *cruzi* and human heart cells that result in cardiac fibrosis, we exposed primary human cardiomyocytes (PHCM) to invasive *T*. *cruzi* trypomastigotes over a two hour time course and evaluated the early changes in gene expression profile that could play a role in the development of cardiac fibrosis observed in Chagas disease. Our results indicate novel genes and kinetics in the expression profile of transcription factors (TFs), cytokines, and chemokines which have been implicated in the development of a fibrogenic phenotype in the heart, liver, kidney and skin in human and animal models. Specifically, our data show that exposure of PHCM to *T*. *cruzi* during a two hour period, time to capture the early events occurring during infection, leads to a significant increase in the transcript levels of JunB, FOS, EGR1, EGR3, and SNAI1; a group of TFs and transcription regulatory genes that have the potential to tilt PHCM towards a profibrotic response. We also show a significant increase in the accumulation of JunB in the nuclear compartment of parasite challenged cells. Furthermore, we show that PHCM respond to *T*. *cruzi* induced activation of profibrotic pathways by upregulating the expression of anti-fibrotic genes including BMP7 and Smad7 in order to potentially control parasite induced fibrogenic gene response. We used our data to develop a profibrotic transcriptome and protein-protein network interactome operating during the initial, early stages of *T*. *cruzi* invasion of PHCMs. Taken together, our findings identify a novel biological interactome and sub-network interactions operating during PHCM—*T*. *cruzi* interaction. A clearer understanding of the initial molecular mechanisms by which *T*. *cruzi* regulates host genes is essential for understanding the cardio-pathology spectrum in Chagas disease. These findings have important implications in defining the pathogenesis of cardiac fibrosis in Chagas heart disease patients.

## Materials and Methods

### Ethics statement

The Institutional Review Board (IRB) at Meharry Medical College approved the study (Control number 081204AAH23163). The IRB granted a waiver to the project because it determined that the project does not require data obtained through intervention, interaction with individuals or identifiable private information.

### Primary human cardiac myocyte culture

Low passage culture of primary human cardiac myocytes (PHCM) was grown according to the manufacturer’s recommendations (PromoCell, Heidelberg, Germany). Briefly, the PHCMs were cultured in myocytes basal growth medium supplemented with the supplemental mix (PromoCell, Heidelberg, Germany) containing fetal calf serum (0.05ml/ml), recombinant human epidermal growth factor (0.5ng/ml), recombinant human basic fibroblast growth factor (2ng/ml) and recombinant human insulin (5ug/ml). The cells were grown in T75 flasks at 37°C in the presence of 5% CO_2_ to a confluence of about 80% (about 4X10^6^ cells) before used in the *T*. *cruzi* infection and control assays.

### Parasite culture and infection assays

Heart myoblast monolayers at 80% confluence, cultured in complete DMEM containing glutamax, 20% fetal bovine serum, 1% each of penicillin/streptomycin, multivitamins and MEM non-essential amino acids (Life Technologies, Carlsbad, CA, USA) were infected with *T*. *cruzi* trypomastigotes. Pure cultures of highly invasive *T*. *cruzi* trypomastigotes clone MMC 20A (Tulahuen strain) were harvested from the supernatant of heart myoblast monolayers as previously described [33,34]. The parasites were washed in Hanks Balanced Salt Solution (HBSS) and resuspended in PHCM growth medium without supplement at 1x10^7^ parasites/ml.

For the infection assays, PHCM confluent monolayers were starved in HBSS containing 30 mM HEPES followed by addition of invasive *T*. *cruzi* trypomastigotes in myocyte basal growth medium without supplements at a ratio of 10 parasites per cell. The cells challenged with the parasites were incubated for different time points 15, 30, 60, 90 and 120 minutes. Each time point was done using three independent T75 flasks for extraction of total RNA, three independent T75 flask for use in the western blotting experiments and three independent sets of 8-well lab-TeK chamber slides for confocal immunofluorescence microscopy assays. Mock-infected (media only) PHCM were used as control for each time point.

### RNA extraction and quality assessment

At each designated time point of the experiment, the parasites were washed off the PHCM cells and the cells were lysed in RNA extraction lysis buffer as described by the manufacturer. Total RNA was purified with the RNAeasy RNA purification kit (Qiagen, Valencia, CA, USA). The RNA purification step included an on column DNase treatment step to eliminate traces of genomic DNA as described by the manufacturer (Qiagen). The quality of the purified RNA was analyzed using the Bioanalyzer 2100 system (Agilent Technologies, Santa Clara, CA, USA). The quality of purified RNA was assessed by RNA Integrity Number (RIN) and samples with a RIN of at least 7 were used for further analysis.

### Microarray hybridization and analysis

Transcriptome microarray analysis were conducted using the Affymetrix GeneChip Human Gene 1.0 ST array which contains ~36,000 NCBI Reference Sequence (RefSeq) transcripts. The microarrary assays were done by Vanderbilt Technologies for Advanced Genomics (VANTAGE) center as described on their website; http://vantage.vanderbilt.edu. Affymetrix CEL files were normalized using the Robust Multi-array Average (RMA) algorithm [35]. Transcriptome level fold change and corresponding significances were analyzed by ANOVA using Partek Genomics Suite (Version 6) at the Meharry Medical College Bioinformatics Core. Significantly changed transcripts were defined as having a ≥ ±2.0 fold expression change from controls and a Benjamini-Hochberg (BH) false discovery rate corrected ANOVA P-value ≤ 0.05. The raw data and processed data have been made available at Gene Expression Omnibus (GEO/NCBI) website (GEO ACCESSION NUMBER GSE75821 and link http://www.ncbi.nlm.nih.gov/geo/query/acc.cgi?acc=GSE75821).

### Biological interaction network and KEGG pathway enrichment analysis

Pathway Enrichment Analysis was performed with WEBGESTALT web analysis software (http://bioinfo.vanderbilt.edu/wg2/) by mapping significantly changed genes to corresponding KEGG pathways and conducting a hypergeometric test for significant enrichment [36–38]. Significance for pathway level enrichment was defined as having an enrichment score False Discovery Rate (FDR) corrected p-value < 0.05. Biological network construction was conducted with the MiMI (Michigan Molecular Interactions) database (29, 30) and the GENEMANIA algorithm [39,40] by querying multiple biological interaction databases including BIND, DIP, HPRD, RefSeq, SwissProt, IPI [41,42]. Transcriptome and qPCR verified genes were set as starting seed nodes, the network was then expanded to one degree of biological interaction using GENEMANIA and visualized with the CYTOSCAPE bioinformatics software platform (http://www.cytoscape.org/) [41,43].

### Quantitative real-time PCR

Quantitative real-time PCR (qPCR) was done to validate the microarray data using a customized PrimePCR assay (Bio-Rad, Hercules, CA, USA) containing the genes of interest. Furthermore, to focus on the transcript of genes and TFs that will play a role in the onset of fibrosis, which is the focus of this manuscript, we used the human fibrosis RT^2^ Profiler PCR Array, PAHS 120D (Qiagen, Valencia, CA, USA). Total RNA (500ng) with a RIN of at least 7 used in the microarray was now converted to cDNA as described by the protocol of either PrimePCR or human fibrosis RT^2^ Profiler PCR Array.

In the PrimePCR array experiment, total RNA (500ng) was converted to cDNA using the iScript cDNA synthesis kit essentially as described by the manufacturer (Bio-Rad, Hercules, CA, USA). The generated cDNA was mixed with SsoAdvanced SYBR green 2X master mix and loaded (20 μl/per well) on a customized PrimePCR plate containing primers for selected genes to be validated including housekeeping genes, and controls as described, www.bio-rad.com/PrimePCR. The PCR amplification was carried out on C1000 Touch Thermal Cycler as described by the manufacturer (Bio-Rad, Hercules, CA, USA). The Ct values of housekeeping genes (beta actin, beta 2 microglobulin and Ribosomal protein, large, P0) were checked for consistency on all plates across all samples. The data was normalized against the house keeping genes and control samples using CFX manager analysis software with support from Bio-Rad technical service using the ΔΔC_T_ method. The primer sequences used for quantification of the transcript levels in the PrimePCR were not disclosed by Bio-Rad.

For the RT^2^ Profiler PCR Array, PAHS 120D (Qiagen, Valencia, CA, USA), an aliquot of 500ng of total RNA was converted to cDNA using RT^2^ First Strand Kit (Qiagen, Valencia, CA, USA). The cDNA was mixed with the RT^2^ SYBR Green Master mix (Qiagen, Valencia, CA, USA), loaded on the PCR array plate and the PCR was run on a C1000 Touch Thermal Cycler (Bio-Rad, Hercules, CA, USA) as described by the manufacturer (www.SABiosciences.com/pcrarrayprotocolfiles.php). The Ct values of housekeeping genes (beta actin, beta 2 microglobulin and Ribosomal protein, large, P0) were checked for consistency on all plates across all samples. The data was normalized against the house keeping genes and control samples using the ΔΔC_T_ method. In all the qPCR assays (PrimePCR and RT^2^ Profiler PCR Array), the sample for each of the three biological replicate was done in duplicate.

### Immunoblotting

Western blot analysis was performed as described previously [44]. Briefly, serum starved PHCM cells (about 80% confluence on a T75 flask) preincubated with *T*. *cruzi* trypomastigotes or media alone were lysed in NP40 Cell Lysis Buffer (Life Technologies, Carlsbad, CA, USA) in the presence of phosphatase inhibitor cocktails 2 and 3 at 1:100 each, (Sigma Aldrich, St. Loius, MO, USA) and protease inhibitor cocktail set III at 1:100, (Calbiochem, Gibbstown, NJ, USA). Whole cell lysates (20μg/lane) were separated by SDS-PAGE on a 4–15% gradient polyacrylamide gel and transferred onto nitrocellulose membranes (Life Technologies, Carlsbad, CA, USA). The membranes were incubated for 1 hour in blocking buffer (1X TBS pH 7.4, 5% nonfat dry milk and 0.1% Tween-20) followed by incubation with goat anti–phospho-Smad2/3 polyclonal antibody (1:225), mouse anti-BMP-7 monoclonal antibody (1:200), rabbit anti-SNAI1 polyclonal antibody (1:250), mouse anti-VDR monoclonal antibody (1:200), rabbit anti-EGR1 antibody 1:200 (Santa Cruz Biotechnology, Santa Cruz, CA, USA), or anti-phospho-SMAD4 antibody 1:250 (Thermo Scientific, Rockford, IL, USA) in blocking buffer at 4°C overnight. The blots were washed and incubated with the corresponding secondary antibody: anti-mouse IgG HRP, anti-rabbit IgG HRP 1:2500 (Promega, Madison, WI, USA) or donkey anti-goat IgG HRP 1:2500 (Santa Cruz Biotechnology) in blocking buffer for 1 hour at room temperature. The blots were washed and the bound antibody revealed using a chemiluminescence kit (Cell Signaling Technology, Danvers, MA, USA) as described by the manufacturer and scanned. The blots were stripped with NewBlot Nitro Stripping Buffer 5X (LI-COR Biosciences, Lincoln, NE, USA) and reprobed with the antibody of a control protein: mouse anti-GAPDH polyclonal antibody (0.5μg/ml, GenScript, Piscataway, NJ, USA), goat anti-beta-actin polyclonal antibody (1:200) or mouse anti-SMAD2 at 1:200 dilution or mouse anti SMAD4 monoclonal antibody at 1:250 (Santa Cruz Biotechnology, Santa Cruz, CA, USA) in blocking buffer for 1 hour at room temp or overnight at 4°C when using SMAD2 or SMAD4 antibody. The blots were washed, probed with corresponding secondary antibody, developed by chemiluminescence and scanned.

The normalized ratio of protein expression level is defined as the ratio of the target protein band intensity to internal control (GAPDH, beta-actin or SMAD2 or SMAD4) protein band intensity, with that of the control sample set as 1.0. Data collected from three independent experiments were analyzed using a student’s t-test where *P<0.05

### Immunofluorescence assays

PHCM cells seeded in Lab-Tek chamber slides were similarly serum starved for 1 hour and exposed to transgenic *T*. *cruzi* expressing Green Fluorescence protein (GFP) at a ratio of 10 parasites per cell for the various time points as discussed earlier. At the end of each time point, the parasites were washed off and cells fixed with 4% paraformaldehyde for 5 minutes at room temp and washed with 1X HBSS. Fixed cells were perforated with 0.1% Triton-X100 in TBS for 5 minutes and blocked with 3% BSA-PBS for 30 minutes at room temperature. Mouse anti human-JunB (C-11) Alexa Fluor-488 IgG antibodies (Santa Cruz Biotechnology) at a dilution of 1:1000, phalloidin (1:2000) were added to the cells and incubated overnight at 4°C. Slides were washed with 1% BSA-PBS. The nucleus was stained with DAPI (1:1000) for 10 minutes at room temp and washed with 1X HBSS. Slides were observed under confocal microscope Nikon A1R at 40X at the Fluorescence Microscopy Morphology Core, Meharry Medical College.

### Multi-Analyte ELISArray

In order to determine if the cytokines/chemokines with increased normalized transcripts were also present in the conditioned media, we used a customized Enzyme Linked Immunosorbent Assay (ELISA) array to quantitate selected cytokines and chemokine in the conditioned media of starved PHCM cells exposed to *T*. *cruzi*. A customized Multi-Analyte ELISArray assay was used to simultaneously profile the kinetics of IL 4, IL 5, IL 6, IL 10, IL 13 and Eotaxin in the conditioned media of PHCM exposed to *T*. *cruzi* as described by the manufacturer (Qiagen). The ELISArray microplates were coated with a panel of our selected target-specific capture antibodies, to obtain qualitative relative profiling data in a sandwich format. Briefly, conditioned cell culture supernatants, positive antigen cocktail and negative control samples were added onto the blocked ELISA plates pre-coated with antibodies evaluated to have the best sensitivity, linearity and lowest background to the selected antigens. The unbound proteins were washed away and biotinylated detection antibodies added to the wells to bind to the captured analyte, and unbound antibodies washed away. The bound biotinylated detection antibodies were detected using an avidin-horseradish peroxidase conjugate and a development substrate. The reaction was stopped and the absorbance read at 450 nm. The reaction was carried out using conditioned media from two biological replicate infection assays and each sample was done in duplicates.

#### Statistical analysis

All experiments were repeated three times. The RT-PCR numerical data are presented as means ± SEM from three biological replicates, unless otherwise indicated, and data were analyzed using two-tailed student’s *t*-test to determine significance. Transcriptome microarray data were the result of three biological replicates at each time point, a two-way ANOVA was used to determine fold change significance and factor out different scan dates of the array chips as a confounding variable. Pathway enrichment analysis used a hypergeometric test with a false discovery rate adjusted p-value to rank the significance of enriched pathways. Statistical significance was defined as *P*<0.05.

## Results

### Transcriptome profile analysis of PHCMs during early *T*. *cruzi* infection

**Fig 1:**
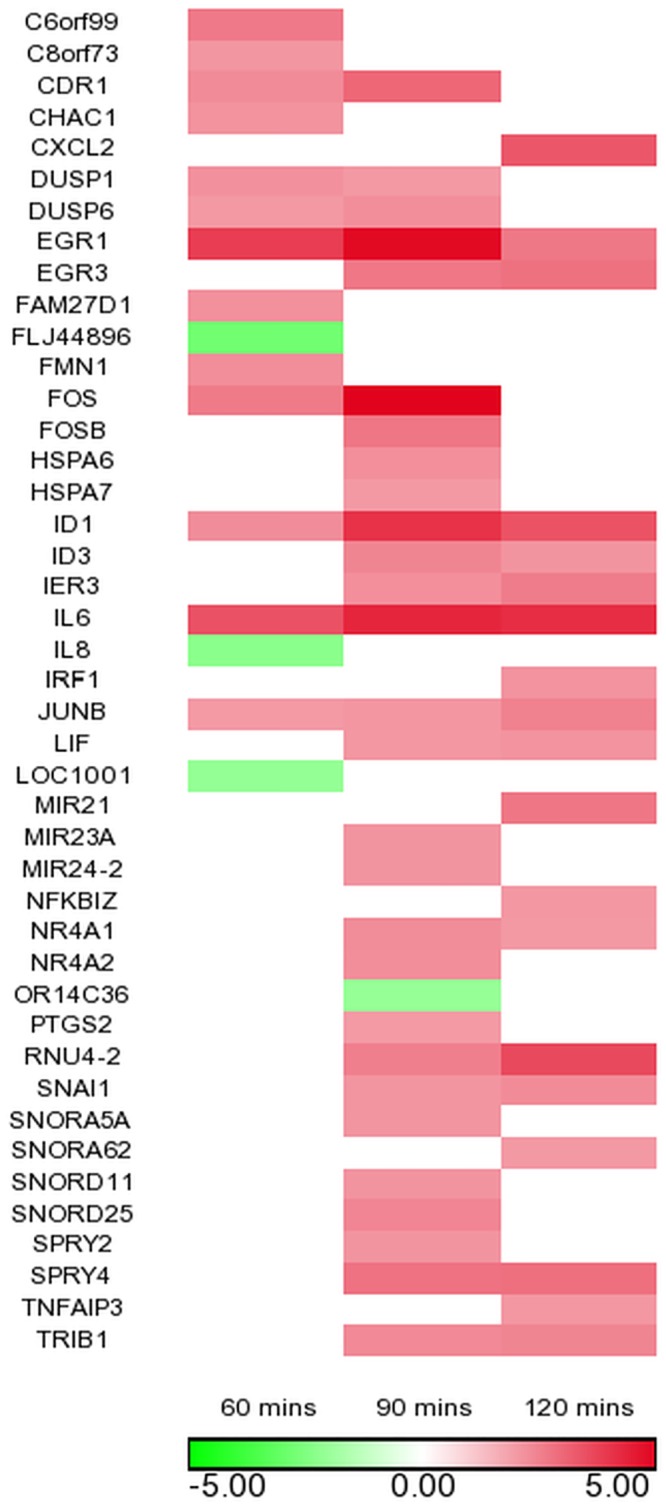
Transcriptome level changes in PHCM genes after exposure to *T*. *cruzi*. Intensity plot of significantly differentially expressed mRNA transcripts in PHCM at 60, 90 and 120 minutes post *T*. *cruzi* exposure shows a strong up-regulation profile of AP-1 transcription factor members (JUNB, FOSB, FOS), EGR family of transcription factors (EGR1, EGR3) and pro-fibrotic cytokines and chemokines (IL5, IL6, IL13, CCL11). Transcriptome profiles were generated from 3 biological replicates at each time point. Significant changes were defined as transcripts having greater than two-fold up or down regulation and a multiple testing corrected ANOVA p-value < 0.05. Red intensity bars show greater than two-fold up regulation while green intensity bars indicate a more than two-fold down-regulation.

In order to identify the genes modulated by *T*. *cruzi* that cause cardiac pathology, we purified total RNA from PHCM exposed to the parasite and used the mRNA to systematically decode the early changes in the gene transcription profile that can play a role in the development of fibrosis. We applied these RNA samples in microarray assays to analyze the changes in the gene expression profile of PHCM, an important cell type that plays a role in cardiac conductivity and function. PHCM were exposed to invasive *T*. *cruzi* trypomastigotes at different time points and the total RNA purified from the challenged PHCM cells was used in our kinetics microarray hybridization assays. This approach represents a novel closest duplication of what happens in the human heart when exposed to the parasite. The arrays were done in biological replicates at different time points and the changes in gene expression greater than 2-fold and having a Benjamini and Hochburg false discovery rate corrected p-value of < 0.05 were considered significant. We evaluated the kinetics of the gene expression profile of PHCM at 60, 90 and 120 minutes, representing the time required for the parasite to bind to and invade host cells, using the Affymetrix HumanGene 1.0 Array platform. We observed that the parasite significantly induced genome wide changes modulating the gene expression profile of several genes as shown in Fig 1. Considering an absolute value expression fold change of > 2 with a false discovery rate (FDR) corrected ANOVA p-value < 0.05 as significant, we observed that the expression of >40 transcripts were significantly changed (Fig 1). Many of the genes upregulated at all the three time points included stress response TFs including EGR1, DNA binding protein inhibitor 1 (ID1), Jun B proto-oncogene (JunB), cytokines including interleukin 6 (IL6) and several other genes that remain to be annotated. We focused our attention to changes in the gene expression profile of TFs that could play a role in the onset of fibrosis or inhibit fibrosis. We used a customized primePCR array assay to confirm the data obtained from the microarray. The quantitative primePCR array confirmed and extended the microarray data as shown in Table 1. Our qPCR data showed that the transcripts of TFs EGR1 and 3, and JunB were upregulated at all-time points while others including SNA1, SMAD7 and CCL-11 were upregulated from 90 minutes of exposure of PHCM to invasive *T*. *cruzi* trypomastigotes (Table 1). The TFs, FOSB and LIF1 shown to be upregulated at all-time points by qPCR were not found to be upregulated at all-time points by the microarray assay (Fig 1 and Table 1).

**Table 1:** Validation of selected fibrotic gene transcripts in PHCM exposed to *T*. *cruzi*.

Gene name and symbol	NCBI RefSeq #	60 mins	90 mins	120 mins
Actin, alpha 2 (ACTA2)	NM_001613	-1.01 ± 0.07	1.58 ± 0.08	1.82 ± 0.07
Angiotensinogen (AGT)	NM_000029	-1.12 ± 0.05	**4.55 ± 0.02**	**3.11 ± 0.09**
Chemokine (C-C motif) ligand 11 (CCL11)	NM_002986	1.88 ± 0.09	**5.58 ± 0.12**	**6.16 ± 0.18**
Connective tissue growth factor (CTGF)	NM_001901	**-2.29 ± 0.02**	**-2.16 ± 0.02**	-1.62 ± 0.08
Interleukin 13 (IL13)	NM_002188	-1.11 ± 0.11	**3.35 ± 0.14**	1.61 ± 0.10
Interleukin 5 (IL5)	NM_000879	1.08 ± 0.01	**2.87 ± 0.04**	1.65 ± 0.09
Snail homolog 1 (SNAI1)	NM_005985	1.61 ± 0.02	**3.70 ± 0.02**	**3.4 ± 0.30**
Early Growth Response 1 (EGR1)	NM_001964	**3.35 ± 0.10**	**6.38 ± 0.11**	**3.65 ± 0.07**
Early Growth Response 3 (EGR3)	NM_004430	**3.12 ± 0.02**	**5.80 ± 0.05**	**4.09 ± 0.07**
FOS	NM_005252	**10.77 ± 0.16**	**25.78 ± 0.09**	1.36 ± 0.14
FOSB	NM_006732	**3.22 ± 0.21**	**7.32 ± 0.14**	**4.31 ± 0.18**
Interleukin 6 (IL 6)	NM_000600	**4.12 ± 0.11**	**6.34 ± 0.96**	**5.14 ± 0.12**
JUNB	NM_002229	**2.10 ± 0.02**	**4.09 ± 0.01**	**4.46 ± 0.04**
SMAD7	NM_005904	1.31 ± 0.05	**2.81 ± 0.08**	**3.24 ± 0.07**
Leukemia inhibitory factor (LIF)	NM_002309	**2.12 ± 0.14**	**4.60 ± 1.07**	**3.73 ± 0.03**
Inhibitor of DNA binding 1 (ID1)	NM_181353	**4.85 ± 0.18**	**15.24 ± 3.48**	**10.99 ± 0.13**
Inhibitor of DNA binding 3 (ID3)	NM_002167	1.62 ± 0.01	**4.05 ± 0.92**	**3.70 ± 0.11**
Dual specificity protein phosphatase 1 (DUSP1)	NM_004417	**2.44 ± 0.04**	**3.15 ± 0.71**	1.78±0.02
Dual specificity protein phosphatase 6 (DUSP6)	NM_001946	1.72 ± 0.02	**3.25 ± 0.74**	**2.04 ± 0.03**

Total RNA purified from serum starved PHCM monolayers exposed to invasive *T*. *cruzi* trypomastigotes used for the microarray assay were also used to validate selected fibrotic gene transcript profiles by qPCR (PrimePCR). The relative expression of each transcript was normalized against RPLPO and beta-2 microglobulin as housekeeping genes. Each value is the mean of biological triplicates performed in technical duplicates ± SEM. The p value for each fold change in the table is less than 0.05. Values of fold-change ≥ 2.0 (in bold) are significantly up-regulated and values of fold-change ≤ -2.0 (in bold) are significantly down-regulated.

### Early *T*. *cruzi* infection regulates PHCM profibrotic genes

**Fig 2:**
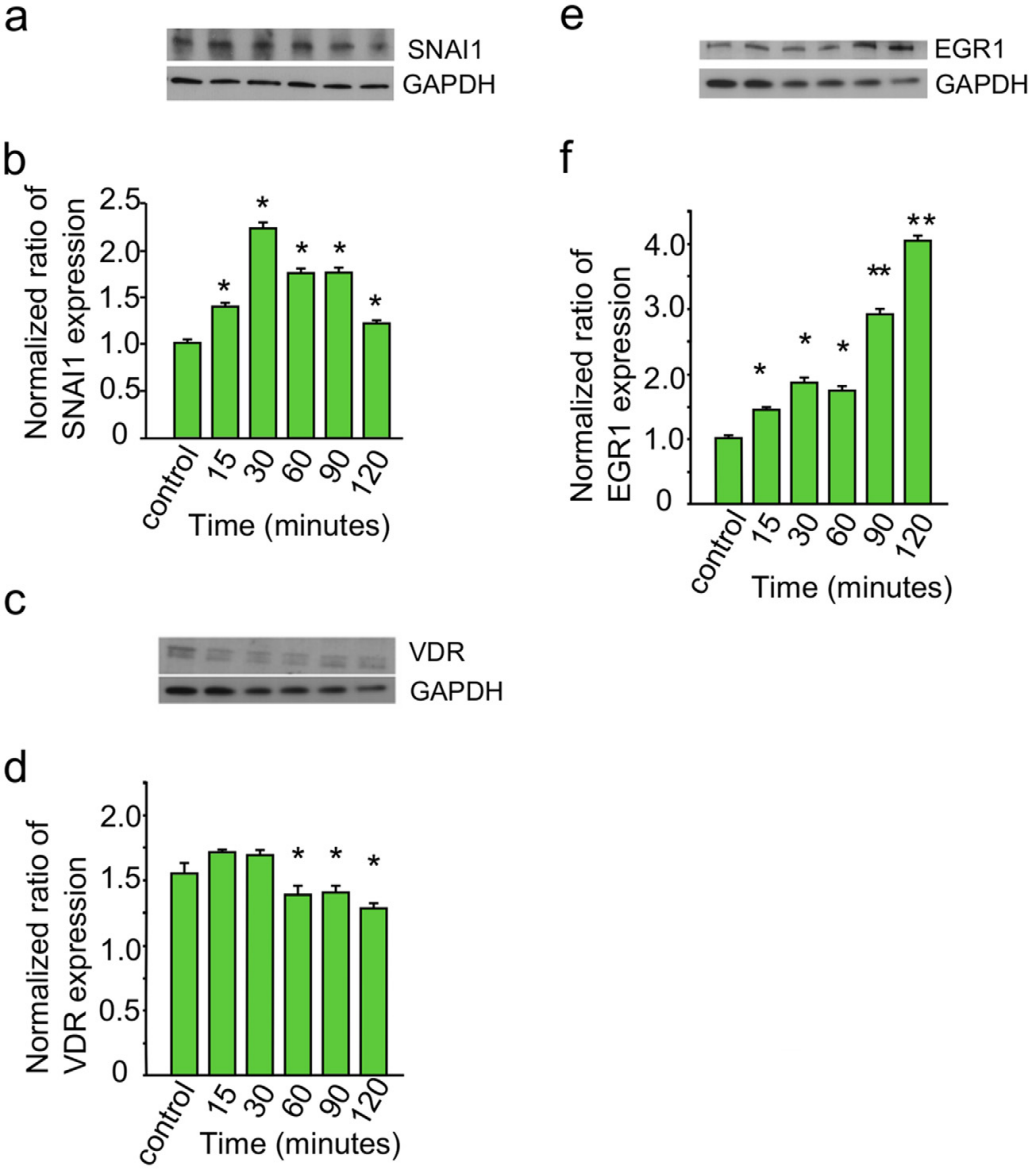
Kinetics of fibrotic gene expression profile of PHCM exposed to *T*. *cruzi*. Serum starved PHCM monolayers exposed to invasive *T*. *cruzi* trypomastigotes at a ratio of 10 parasites/cell at different time points were used for evaluating protein levels of profibrotic TF by western blot assays. Cell lysates of PHCM exposed to *T*. *cruzi* at different time points were separated by SDS-PAGE, transferred to NC membrane, probed with (a) anti-SNAI1 antibody and developed by chemiluminescence. Blot showing the bands for SNAI1 and GAPDH is a representative of 3 independent experiments conducted with similar results. (b) The graph summarizes a densitometric analysis of 3 independent experiments showing SNAI1 protein expression kinetics normalized against GAPDH. (c) Cell lysate blots were probed with VDR antibody and developed by chemiluminescence. Blot showing the bands for VDR and GAPDH is a representative of 3 independent experiments conducted with similar results. (d) Column graph summarizes densitometric analysis of 3 independent experiments showing VDR protein expression kinetics normalized against GAPDH. (e) Cell lysates were also probed with EGR1 antibody and developed by chemiluminescence. Blot showing the bands for EGR1 and GAPDH is a representative of 3 independent experiments conducted with similar results. (f) Column graph summarizes densitometric analysis of 3 independent experiments showing EGR1 protein expression kinetics normalized against GAPDH. *P<0.05 vs control, **P<0.01 vs control. The data on each column graph (b, d and f) show a representative experiment of 3 independent experiments performed with similar results.

In order to look deeper into the modulation of profibrotic genes by *T*. *cruzi* in PHCM early during the process of cellular infection, we used the fibrotic RT^2^ Profiler PCR array assay as another approach to evaluate the transcription profile of profibrotic genes. We observed that *T*. *cruzi* modulated the expression of several profibrotic genes early during the process of infection. The microarray and PCR array showed that *T*. *cruzi* was able to regulate the transcripts levels of profibrotic TFs, inflammatory chemokines and cytokines. The normalized fold regulation of SNAI1 transcript is significantly upregulated at 90 and 120 minutes in the microarray and both qPCR assays (Fig 1, Table 1 and S1 Table). To further examine the changes in gene expression, we looked at the post-transcriptional level to check whether the protein expression level of SNAI1 was also increased to complement the increase in SNAI1 transcript. We used immunoblotting to evaluate the amount of SNA1 protein in PHCM cell lysate exposed to invasive *T*. *cruzi* at 15, 30, 60, 90 and 120 minutes. We observed that the normalized ratio of SNAI1 protein expression was significantly increased up to a maximum of 2.3±0.02 fold at 30 minutes and gradually decreased to 1.3±0.01 fold at 120 minutes compared to control (Fig 2a and 2b). This indicates that *T*. *cruzi* upregulates the expression of both the transcript and the protein expression levels of SNAI1 in cardiac myocytes early during the process of infection. An effective upregulation of SNAI1 is usually accompanied by changes in the expression of vitamin D receptor (VDR) protein downstream. To verify if the protein level of VDR was affected in the cells following SNAI1 upregulation, we reprobed the blots with a VDR antibody and a house keeping gene for quantification (Fig 2c and 2d). Normalization of the scanned blot with the house keeping gene (GAPDH) showed that the ratio of VDR protein expression exhibited a significant gradual decrease from 1.1±0.01 at 15 minutes to 0.8±0.02 at 120 minutes in HPCM exposed to *T*. *cruzi* trypomastigotes (Fig 2c and 2d).

Another important profibrotic response TF gene which was significantly upregulated at all-time points in the microarray assay and validated by qPCR is EGR1. EGR1 regulates expression of growth factors, cytokines and other TFs including leukemia inhibitory factor (LIF) that was also upregulated at all-time points in the quantitative real-time PCR assays. Therefore, we decided to evaluate the protein expression levels of EGR1 in the lysate of PHCM exposed to *T*. *cruzi* to determine if the validated increase in transcript levels matches a corresponding increase in the protein level. Antibodies to EGR1 and the house keeping gene GAPDH were used to probe the challenged and control cell lysate blots (Fig 2e and 2f). Analysis of the normalized ratio of EGR1 protein expression compared to the house keeping gene showed that EGR1 protein level exhibited a significant continuous increase from 1.4±0.04 at 15 minutes to 4.0±0.09 at 120 minutes as shown in the bar graph (Fig 2g).

### Regulation and balance of early fibrotic response in PHCM

**Fig 3:**
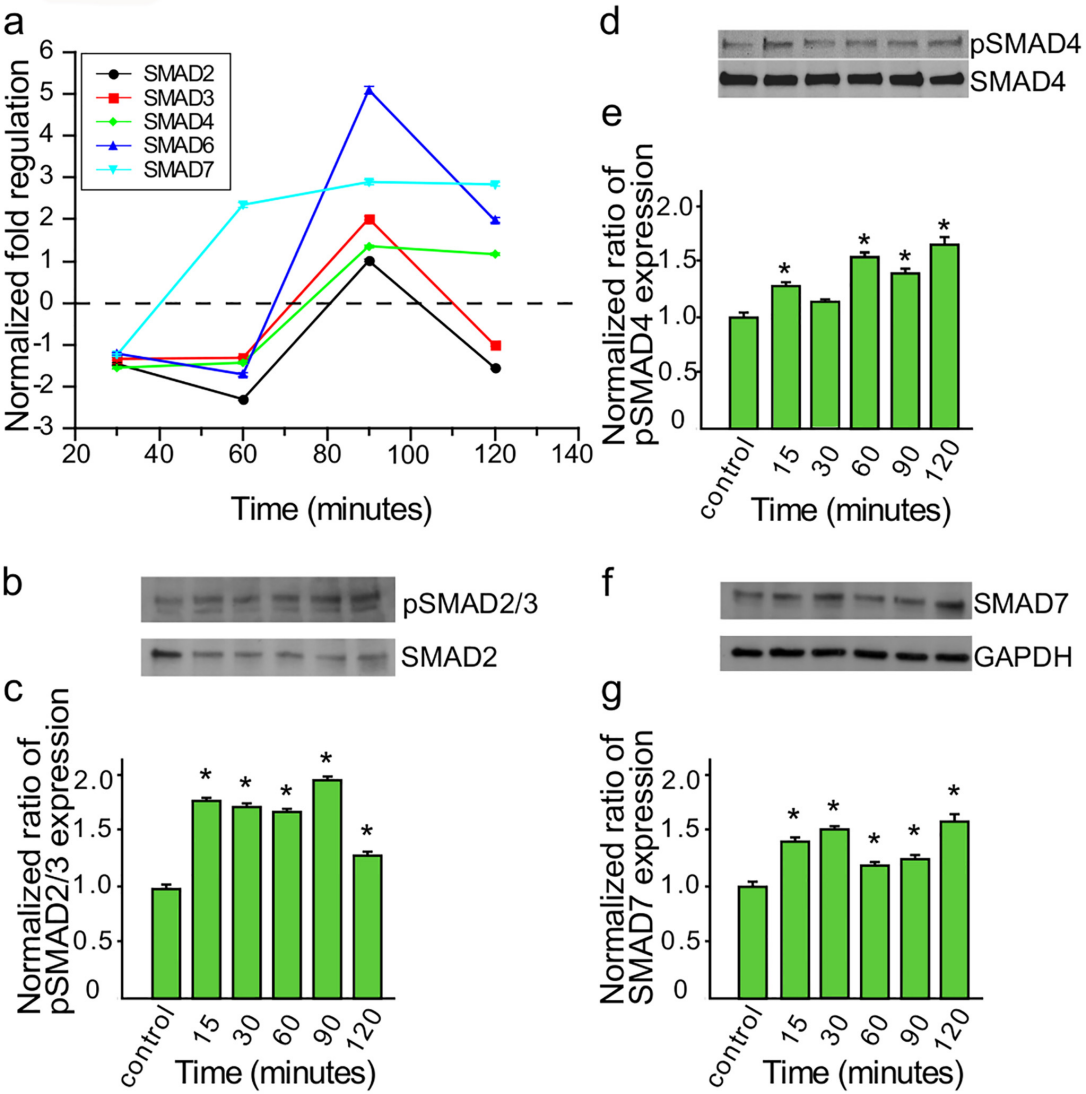
Invasive *T*. *cruzi* trypomastigotes up regulate the expression of SMAD genes in PHCM. Serum starved PHCM monolayers exposed to invasive *T*. *cruzi* trypomastigotes were used for evaluating gene transcript profile by PCR arrays and TF protein kinetics by western blot assays. (a) Total RNA (500ng) purified from the cells were converted to cDNA and used for SMAD gene transcript quantitation using the fibrosis RT^2^ Profiler PCR Array. Line graph shows the mean±SEM of normalized fold regulation of each of the SMAD genes in the PCR array at different time points. (b) Cell lysates of PHCM exposed to *T*. *cruzi* at different time points were separated by SDS-PAGE, transferred to NC membrane, probed with anti pSMAD2/3 antibody and developed by chemiluminescence, stripped and reprobed with SMAD2 antibody. Blots showing the bands for pSMAD2/3 and SMAD2 are a representative of three independent experiments conducted with similar results. (c) Column graph summarizes densitometric analysis of 3 independent experiments showing pSMAD2/3 protein kinetics normalized against SMAD2. *P< 0.05 vs controls. (d) Cell lysate blots were also probed with pSMAD4 antibody and developed by chemiluminescence, stripped and reprobed with SMAD4 antibody. Blots showing the bands for pSMAD4 and SMAD4 are a representative of three independent experiments conducted with similar results. (e) Column graph summarizes densitometric analysis of 3 independent experiments showing pSMAD4 protein expression kinetics normalized against SMAD4. *P< 0.05 vs controls. (f) Cell lysate blots were also probed with SMAD7 antibody and developed by chemiluminescence. Blots showing the bands for SMAD7 and GAPDH are a representative of three independent experiments conducted with similar results. (g) Column graph summarizes densitometric analysis of 3 independent experiments showing SMAD7 protein expression kinetics normalized against GAPDH. *P< 0.05 vs controls.

Transforming growth factor-beta (TGF-β) is a profibrotic cytokine that plays a critical role in the development of tissue fibrosis through activated SMAD family of TFs. The SMAD family of TFs is at the core of the TGF-β pathway which can also be blocked by inhibitory SMADs (SMAD6 and SAMD7) that antagonize the signaling function of the other SMADS. Therefore we looked at the transcription profiles and protein levels of phosphorylated SMAD2/3 (pSMAD2/3). In the transcript evaluation assays validated by customized PrimePCR and RT^2^ Profiler PCR Array, SMAD7 was significantly upregulated at 90 minutes (2.8±0.08 normalized fold change) and 120 minutes (3.2±0.07 normalized fold change) in the PrimePCR assays (Table 1) and it was found to be upregulated from 60 to 120 minutes in the PCR array (Fig 3a). SMADs 2, 3 and 4 transcripts did not show any significant changes at any time point in all three assays (microarray, PrimePCR and PCR array). Since pSMAD2/3 is the activated state which can be translocated into the nucleus in a complex with SMAD4, we decided to evaluate the level of pSMAD2/3 in the cell lysate of PHCM cells exposed to *T*. *cruzi* at different time points (Fig 3b and 3c). We observed that the ratio of pSMAD2/3 normalized against total SMAD2 remained significantly increased when the PHCM were exposed to *T*. *cruzi* to a maximum of 1.9±0.021 at 90 minutes (Fig 3c). We also checked the protein expression levels of the co-effector pSMAD4 compared to total SMAD4 and we observed that normalized ratio of pSMAD4 expression significantly increased at 15 minutes to 1.31±0.02, decreased at 30 minutes to 1.15±0.04 and again increased to a maximum of 1.7±0.05 at 120 minutes (Fig 3d and 3e). The pSMAD4 expression showed a general increase with increase in time of exposure of PHCM to *T*. *cruzi* trypomastigotes. In addition, the protein expression level of the inhibitory SMAD7 in PHCM exposed to the parasites normalized against GAPDH and the control PHCM was also significantly upregulated to a maximum value of 1.6±0.05 at 120 minutes (Fig 3f and 3g).

**Fig 4:**
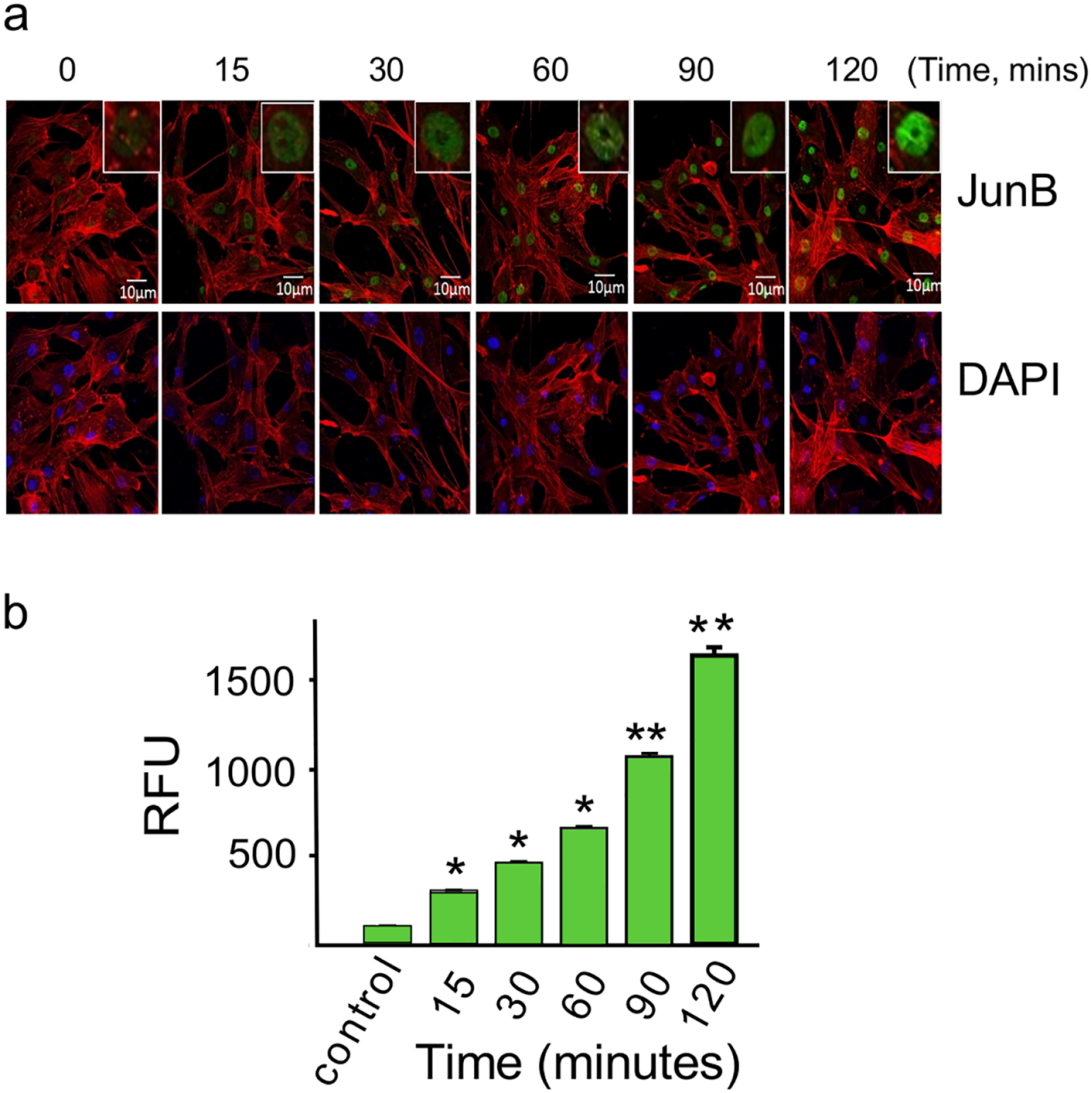
Invasive *T*. *cruzi* trypomastigotes upregulates nuclear translocation of JunB in PHCM. PHCM seeded onto Lab-Tek chamber slides were serum starved and exposed to transgenic *T*. *cruzi* expressing GFP at a ratio of 10 parasites/cell at different time points followed by washing with PBS. Cells were probed with mouse anti human-JunB Alexa Fluor-488 IgG (green), actin myofibrils were stained red with phalloidin, nuclei stained blue with DAPI and visualized by Confocal microscopy using 40X magnification. Panel (a), upper panel shows an increase in green fluorescent staining of JunB with time. Lower panel shows DAPI and phalloidin staining. (b) Column graph shows normalized quantitative measurement of fluorescence intensity using CAStream software. The fluorescence intensity was normalized with the control and plotted. This is a representative experiment of three independent experiments performed with similar results. *P<0.05 vs control, **P<0.01 vs control.

We used confocal microscopy to determine whether JunB, a component of the AP-1 complex showing a significantly increased transcript levels at all time points investigated, was also translocated to the nucleus when PHCM is challenged with the parasite. We observed that the RFU of JunB stained in the nuclei of challenged cells significantly increased with time (Fig 4a and 4b). The RFU of JunB stained in the nucleus increased significantly from 3015±94 RFU at 15 minutes continuously to a maximum of 16300±148 RFU at 120 minutes (Fig 4a and 4b).

In order to evaluate selected cytokines and chemokine in the conditioned media from PHCM exposed to *T*. *cruzi* trypomastigotes, we employed a multi-analyte ELISArray to determine the relative abundance of the cytokines and chemokine with a significantly increased transcript level in the media. We observed that only IL6 was significantly secreted in the conditioned media to a maximum relative absorbance of 0.319±0.031 in the conditioned of PHCM exposed to the parasites for 120 minutes (Table 2).

**Table 2:** Expression of cytokines/chemokines in PHCM conditioned media.

	Absorbance at 450 nm					
Time (mins)	IL 4	IL 5	IL 6	IL 10	IL 13	Eotaxin
0	0.0415±0.009	0.047±0.001	0.058±0.004	0.0455±0.002	0.057±0.006	0.2095±0.219
15	0.0465±0.002	0.0425±0.001	0.07±0.035	0.046±0.001	0.0485±0.005	0.05±0.001
30	0.0505±0.001	0.054±0.001	0.1575±0.005	0.0575±0.009	0.0505±0.002	0.0545±0.001
60	0.05±0.004	0.046±0.0014	0.072±0.003	0.0465±0.001	0.052±0.003	0.0515±0.004
90	0.0465±0.001	0.048±0.001	0.249±0.016	0.0495±0.002	0.053±0.001	0.056±0.001
120	0.0445±0.001	0.046±0.001	0.319±0.031	0.041±0.006	0.0495±0.001	0.053±0.001

Conditioned media from serum starved PHCM monolayers exposed to invasive *T*. *cruzi* trypomastigotes was used for detection of cytokines/chemokines using a multi-analyte sandwich ELISArray. Each value is the mean absorbance at 450 nm of biological replicates performed in duplicates ± SEM.

### Early *T*. *cruzi* infection regulates anti-fibrotic PHCM genes

**Fig 5:**
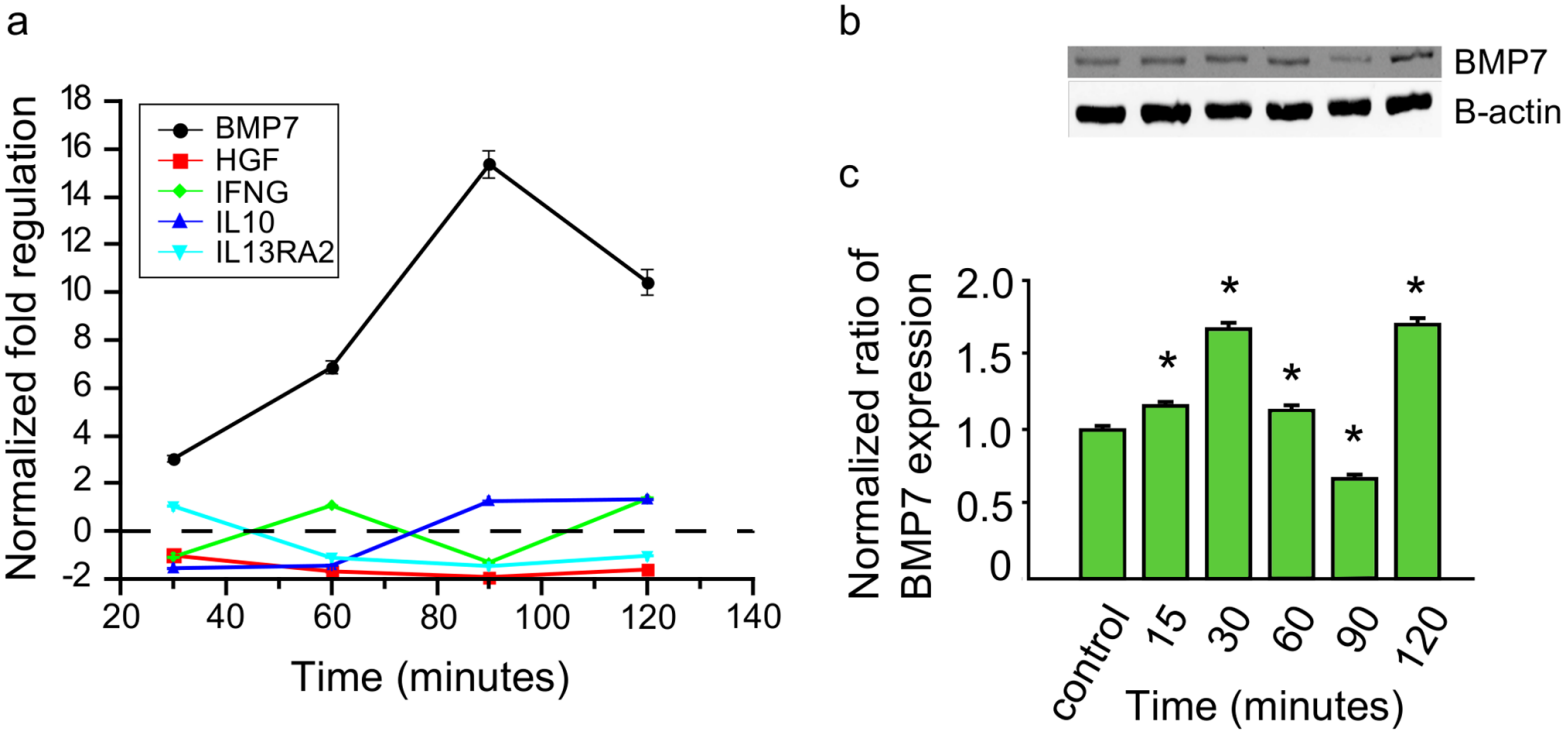
Invasive *T*. *cruzi* trypomastigotes up regulate the expression of antifibrotic genes in PHCM. Serum starved PHCM monolayers exposed to invasive *T*. *cruzi* trypomastigotes at different time points were used for evaluating the fibrotic gene transcription profile by PCR array and western blot assays. (a) Total RNA (500ng) purified from the cells were converted to cDNA and used for antifibrotic gene transcript quantitation by fibrosis RT^2^ Profiler PCR Arrays. Line graph shows the mean±SEM (n = 3) of normalized fold regulation of each of the designated antifibrotic genes at different time points. (b) Cell lysates of PHCM exposed to *T*. *cruzi* at different time points were separated by SDS-PAGE, transferred to NC membrane, probed with anti BMP7 antibody and developed by chemiluminescence. Blots showing the bands for BMP7 and β-actin are a representative of three independent experiments conducted with similar results. (C) Column graph summarizes densitometric analysis of 3 independent experiments showing BMP7 protein expression kinetics normalized against β -actin. *P< 0.05 vs controls.

Our observation that *T*. *cruzi* upregulated the transcript and protein level of profibrotic genes, prompted us to also evaluate the regulation of anti-fibrotic genes represented on the PCR array. We observed that the parasite significantly induced the upregulation of the normalized transcript level of bone morphogenic protein 7 (BMP7) to a maximum of 15.3±0.18 at 90 minutes before decreasing to 10.4±0.2 at 120 minutes (Fig 5a). The transcript level of the other anti-fibrotic genes on the array remained unchanged. Furthermore we checked at the protein level to evaluate the kinetics of the normalized BMP7 protein in total cell lysate of PHCM exposed to the parasite. We found that the normalized ratio of BMP7 protein expression was significantly increased by the parasite to 1.6±0.09 at 30 minutes, decreased to 0.68±0.01 at 90 minutes then increased again to 1.7±0.02 at 120 minutes (Fig 5b and 5c).

### *T*. *cruzi* modulates several AP1 containing signaling pathways

**Fig 6:**
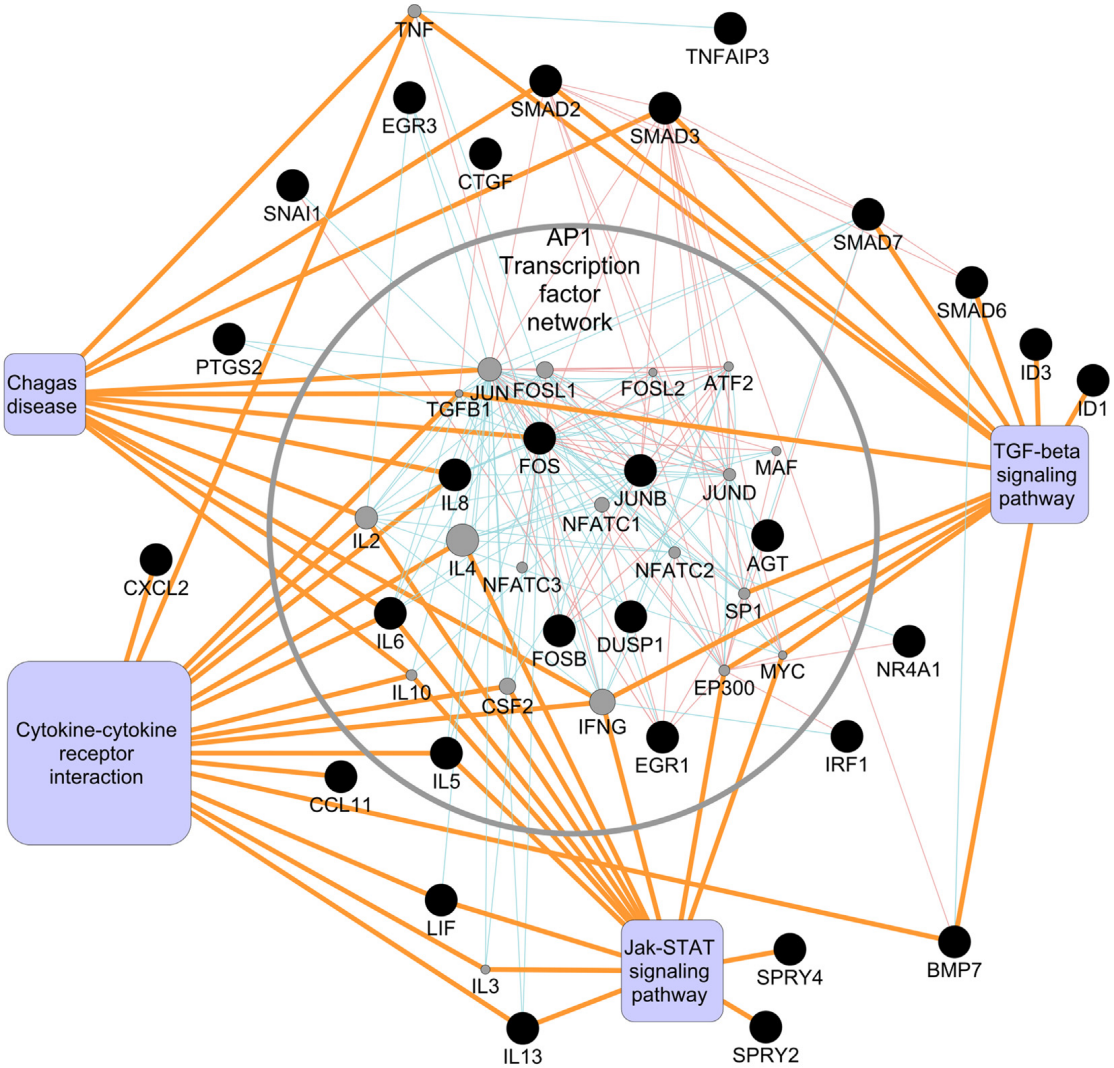
Biological interactions and connectivity network of PHCM transcripts regulated by *T*. *cruzi*. A biological interaction network was created using significantly changed transcriptome microarray and qPCR genes as primary seed nodes for querying multiple biological interaction and pathway centric databases out to one degree of interaction. Primary seed nodes are shown as black circles, interaction expansion nodes (gray circles) were added to the network based on confirmed interactions with at least two primary seed nodes. Red/pink connections (edges) between nodes denote interactions based on physical interactions database sources such as BIND, MINT and DIP. Blue edges between nodes denote pathway data base derived interactions from KEGG, REACTOME, BIOCARTA and the NCI Pathway Interaction Database. Orange edges denote nodes mapping to their associated KEGG pathways.

**Fig 7:**
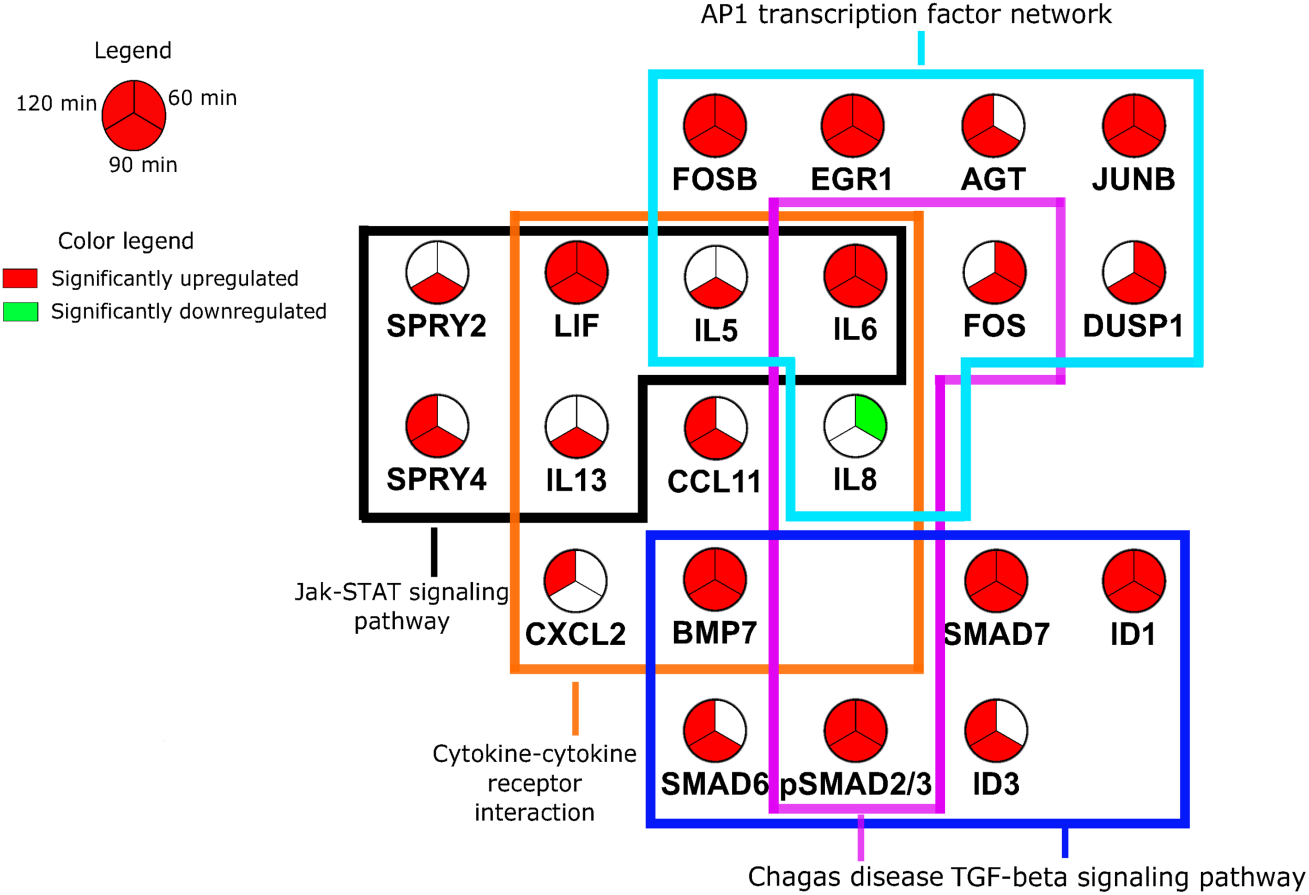
Pathway intersection graph of genes modulated by *T*. *cruzi* in PHCM. Transcriptome array and Real Time-PCR genes mapping to significantly enriched pathways and interactions groups show a highly connected and overlapping structure. Enriched KEGG pathways (TGF-β signaling, Jak-STAT signaling, Cytokine-cytokine receptor interaction and Chagas disease) share multiple genes between them as well as with the AP1 transcriptional factor network. Gene nodes are sectioned into three slices to denote significant expression changes at the 60, 90 and 120 minute time points. Pie slices in red denote a greater than two-fold up regulation, slices in green denote less than a negative two-fold down regulation.

In order to systematically decode the early cellular mechanisms triggered when *T*. *cruzi* comes in contact with PHCM, we used a biological interaction approach to identify potential protein-protein interactions as well as pathway mapping to construct molecular network based view of this process. This network was mapped using the host genes which were significantly modulated by *T*. *cruzi* in the microarray and qPCR data to give us insight into the protein-protein and pathway level interaction networks occurring early in the human heart during *T*. *cruzi* infection. Pathway enrichment analysis, conducted using KEGG (Kyoto Encyclopedia of Genes and Genomes) [45–47] as a mapping database, revealed multiple points of commonality and overlap among the Cytokine-cytokine receptor interaction pathway (hsa04360), Jak-STAT signaling (hsa04060), MAPK signaling (hsa04010) and the TGF-β signaling pathways (hsa04350) as the most significantly enriched pathways (Table 3). Using the pathway enrichment as a basis for creating a biological interactions map, the genes corresponding to the pathway results were used as seed base nodes and the interactions map was expanded to one degree of biological connectivity using the ENRICHMENT MAP database as shown in Fig 6 [48]. The ENRICHMENT MAP project aggregates multiple gene set enrichment interactions and pathway databases, including KEGG, BIOCARTA, REACTOME and the NCI PATHWAY INTERACTIONS DATABASE in order to provide a high level view of biological connectivity. This allowed for inclusion of protein level knowledge in addition to the transcriptome results that were initially obtained, providing a more validated view of the initiation of a fibrotic process in *T*. *cruzi* infection of PHCM cells. With this approach, we noted that JUN, IFN-γ and TGF-β proteins were highly connected at the protein-protein interaction (PPI) level and this observation effectively bridged transcriptome data to our protein data. The intersections of multiple pathways and the shared genes/proteins within them provide a multi-dimensional systems view with a point of convergence, in particular with the AP1 transcription factor network being a central hub for cross-signaling events between the TGF-β signaling pathway, Cytokine-cytokine receptor interactions and Chagas disease pathways (Figs 6 and 7). Additionally, Jak-STAT signaling pathway involvement is in agreement with previous observations suggesting that it is critically involved in the pathogenesis of cardiac remodeling through cytokine signaling (Table 3) and angiotensinogen [49]. Of note is the fact that 11 significantly altered genes or proteins from our dataset were members of more than one of the enriched signaling pathways, thus demonstrating a highly interconnected and complex cascade of events at the transcriptional and proteomic level upon *T*. *cruzi* infection. This cascade of events can lead to the induction of a fibrotic response which progresses to Chagas’ heart disease.

**Table 3:** Pathway Enrichment analysis of significantly changed genes.

KEGG Pathway Name	Pathway Rank	GENES	FDR adjusted enrichment score p-value
TGF-beta signaling pathway (hsa04350)	1	SMAD3, SMAD2, SMAD6, SMAD7, ID1, ID3, BMP7	3.19E-10
Cytokine-cytokine receptor interaction (hsa04630)	2	LIF, CXCL2, CCL11, IL6, IL5, IL8, IL13, BMP7	1.73E-08
Jak-STAT signaling pathway (hsa04060)	3	LIF, SPRY4, SPRY2, IL6, IL5, IL13	2.65E-07
Chagas disease (American trypanosomiasis) (hsa05142)	4	SMAD2, SMAD3, FOS, IL6, IL8	1.31e-06
NOD-like receptor signaling pathway (hsa04621)	5	CXCL2, TNFAIP3, IL6, IL8	4.43E-06

Significantly changed transcriptome array and Real Time—PCR validated genes were mapped to their corresponding KEGG pathways in order to perform cell signaling pathway based enrichment analysis. Column 1 lists the canonical KEGG pathway name with its reference number in parenthesis, column 2 lists the pathway enrichment score rank in terms of p-value determined by hypergeometric test, column 3 lists the genes that mapped to the KEGG pathway and column 4 shows the FDR adjusted p-value of significance of the pathway enrichment score.

## Discussion

Previous studies from our laboratory showed that *T*. *cruzi* regulates extracellular matrix (ECM) interactome and uses it to facilitate the process of cellular infection [50–52]. The regulation of the ECM by the parasite needs to maintain a fine balance between synthesis and degradation of ECM proteins to prevent the accumulation of ECM proteins leading to a fibrotic response which can cause ventricular arrhythmias [6,53]. The mechanism by which *T*. *cruzi* causes accumulation of ECM proteins leading to cardiac fibrosis remains unknown despite several advances in the field. Furthermore, the gene and protein-protein interaction network operating during *T*. *cruzi* infection that favors the onset of fibrosis also remains to be elucidated. Other pathogens have also been implicated in the development of tissue and organ fibrosis. Adenoviruses and enteroviruses such as the coxsackieviruses have been implicated in the development of myocarditis [54]. Premature expression of profibrotic genes have been observed in the muscle of HIV infected patients [55]. In the present study, we exposed invasive *T*. *cruzi* trypomastigotes to primary human heart cells for a period of two hours to study the early molecular changes induced by the parasite that are potentially profibrotic eventually leading to a compromised heart structure and function. This data generated using PHCM gives a close approximation to what could be operating during *T*. *cruzi* infection which naturally causes heart disease making it a good model system to study the natural onset of cardiac fibrosis. The elucidation of the molecular mechanism of *T*. *cruzi*-PHCM interaction will contribute to a better understanding of the pathogenesis of Chagas disease [56]. We focused our attention here to the TFs, chemokines and cytokines regulated by the parasite in PHCM because precise delineation of the major TFs and inflammatory molecules that are modulated early during *T*. *cruzi*-PHCM interaction is essential for understanding the molecular mechanism of consequent fibrosis induced by this interaction downstream. Our data shows the genes regulated by the parasite in PHCM independent of other heart cells which are also infected by the parasite including cardiac fibroblast, immune cells and the overall cardiac microenvironment which will also play a role in the final fibrotic picture observed in Chagas heart disease patients. Currently, we are studying how *T*. *cruzi* regulates gene profiling in the major types of primary human heart cells.

We observed that *T*. *cruzi* upregulates the transcript level of SMAD7 compared to SMAD2/3. The parasite enhanced the phosphorylation of SMAD2/3 (Fig 3) without increasing their transcript level. Others suggested that pSMAD2/3 forms a complex with SMAD4 for translocation into the nucleus where it binds to Smad-response elements (SRE) to regulate gene transcription of several profibrotic genes [25,57]. That is in agreement with our data which shows that there is an accompanied increase in the level of pSMAD4 with time. Activated SMAD2/3 constitutes part of TGF-β downstream signaling molecules which have been suggested to play a role in the development of a fibrotic phenotype [25,58]. Our observed increased in the level of pSMAD during *T*. *cruzi*-PHCM interaction corroborates previous findings where pSMAD2 was reported in the cardiomyocytes from Chagas disease patients [27]. Others also showed that the upregulated TGF-β pathway plays a role in ECM deposition in non-*T*. *cruzi* infected cells indicating the significance of a paracrine effects in the development of Chagas heart disease [8,19]. The PHCM exposed to *T*. *cruzi* also had increased transcript and protein levels of inhibitory SMAD7 at the same time in an attempt to prevent the early initiation of a profibrotic response. We showed that the transcript level of angiotensinogen in PHCM exposed to the parasite is significantly increased. Angiotensinogen is processed to angiotensin II which increases TGF-β playing an important role in the pathophysiology of cardiac fibrosis, heart failure and glomerulosclerosis in humans [59,60].

Furthermore, we observed that the parasite significantly increased the transcript level of EGR1 and EGR3, and the protein level of EGR1. EGR1 is essential for EMT leading to fibrosis through upregulation of SNAI1 [61] which was also increased in our studies at both the transcript and protein levels. SNAI1 upregulation was accompanied by a concomitant decreased protein expression of downstream VDR. This observation is in line with the suggestion that vitamin D deficiency is associated with cardiovascular disease [62]. It is not known how changes in VDR level of protein expression, activity and polymorphisms relates to the development of cardiac pathology in individuals presenting with Chagas heart disease. The increased in the transcript level of EGR1 and dual specificity protein phosphatase 6 (DUSP6) is in agreement with others who challenged epithelial cells with *T*. *cruzi* [63]. EGR1 expression was shown to be aberrantly increased in hypertrophied hearts of mice treated with hypertrophy inducing agents [64,65] and the absence of EGR1 resulted in a blunted response to hypertrophy-inducing agents in the heart [66]. These observations and ours suggest that EGR1 which is upregulated early in PHCM during *T*. *cruzi* infection plays an important role in the development of cardiac fibrosis.

Our microarray and quantitative PCR assays also showed that *T*. *cruzi* significantly upregulated transcript levels of chemokines like CCL-11 previously shown to correlate with myocardial fibrosis [67] and inflammatory cytokines that play a role in the pathogenesis of fibrosis. It was suggested in a mouse model that IL-13 activates liver fibrosis in a mechanism that is independent of TGF-β showing the importance of this cytokine in the potential management of fibrosis [68]. Th2 cytokines IL-4, IL-5 and IL-13 are dramatically increased in liver fibrosis with IL-13 being an indispensable mediator of fibrosis [69]. The upregulation of IL-5 and IL-13 transcripts in our *T*. *cruzi*-PHCM interaction assay suggest that the parasite might be using both TGF-β dependent and independent pathways to induce cardiac fibrosis, pathways that we are currently investigating in our laboratory. In our *in vitro* assay, we observed that IL-6 is present in the conditioned media of PHCM exposed to *T*. *cruzi* compared to the other cytokines and chemokine tested. Our finding supports the observation suggesting that IL-6 elevations *in vivo* induce cardiac fibrosis [70]. IL-4 was also reported to be upregulated in cardiac fibrosis following aortic coarctation in mice, an observation that was attenuated with IL-4 neutralizing antibody suggesting that the cytokine plays a role in the development of cardiac fibrosis [71]. The cell lysate of PHCM incubated with *T*. *cruzi* showed an increase in the expression of anti-fibrotic TF SMAD7 which can block the action of the phosphoSMAD2/3 proteins. Furthermore, bone morphogenic protein 7 (BMP7) shown to be upregulated can counteract the ongoing TGF-β like fibrotic response being induced by the parasite [23]. Since the profibrotic response players are much more than the anti-fibrotic players, it seems more likely that overtime, the gene transcription profile of the PHCM will eventually tilt towards a profibrotic phenotype. We also observed that the transcript level of components of AP-1 including FOS, FOSB and JunB were significantly upregulated by *T*. *cruzi* during all the early time points considered in our experiments. We used confocal fluorescence microscopy to show that JunB protein is translocated into the nucleus of PHCM exposed to *T*. *cruzi* and the amount of JunB translocated into the nucleus increases significantly with time. Our observation supports previous reports suggesting that during the development of cardiac fibrosis, AP-1 is activated to turn on profibrotic pathways [32,72]. Taken together, our data shows that *T*. *cruzi* initiates a profibrotic response in PHCM early during the process of cellular infection. The parasite induces an increase in the transcript and protein levels of AP-1 complex component JunB and EMT TFs to cause heart disease observed in Chagas heart disease. The identification of AP-1 TF network as the central hub in the early *T*. *cruzi* induced fibrotic response makes it an attractive starting point for the development of small molecule inhibitors for the management of downstream *T*. *cruzi* induced cardiomyopathy. Furthermore, the novel early genetic and proteomic changes reported in our study could be exploited in the development of biomarkers for the identification of *T*. *cruzi* infected subpopulations at risk developing *T*. *cruzi*–induced cardiomyopathy.

## Supporting Information

S1 Table Fibrotic gene transcript profile in PHCM exposed to *T*. *cruzi*. Total RNA purified from serum starved PHCM monolayers exposed to invasive *T*. *cruzi* trypomastigotes used for the microarray assay were also used in a fibrotic PCR array assay to evaluate gene transcript profiles by qPCR. Total RNA (500ng) purified from the cells were converted to cDNA and used for antifibrotic gene transcript quantitation by fibrosis RT^2^ Profiler PCR Arrays. The relative expression of each transcript was normalized against housekeeping genes. Each value is the mean of biological triplicates performed in technical duplicates. The p value for each fold change in the table is less than 0.05.
(DOCX)
