# Supplementary material for: Early Regulation of Profibrotic Genes in Primary Human Cardiac Myocytes by Trypanosoma cruzi
Source: PLoS Negl Trop Dis. 2016 Jan 15;10(1):e0003747. doi: 10.1371/journal.pntd.0003747 (PMC4714843; doi:10.1371/journal.pntd.0003747)
Supplement: S1 Table — Total RNA purified from serum starved PHCM monolayers exposed to invasive T. cruzi trypomastigotes used for the microarray assay were also used in a fibrotic PCR array assay to evaluate gene transcript profiles by qPCR. Total RNA (500ng) purified from the cells were converted to cDNA and used for antifibrotic gene transcript quantitation by fibrosis RT2 Profiler PCR Arrays. The relative expression of each transcript was normalized against housekeeping genes. Each value is the mean of biological triplicates performed in technical duplicates. The p value for each fold change in the table is less than 0.05. (DOCX) [file pntd.0003747.s001.docx]

|  |  | **30 mins** | **60 mins** | **90 mins** | **120 mins** |
| --- | --- | --- | --- | --- | --- |
| **Symbol** | **GeneBank No.** | **Fold Regulation** | **Fold Regulation** | **Fold Regulation** | **Fold Regulation** |
| ACTA2 | NM_001613 | -1.1503 | -1.0092 | -1.5782 | 1.8155 |
| AGT | NM_000029 | -1.4816 | -1.1222 | 4.5503 | 3.0987 |
| AKT1 | NM_005163 | 1.1367 | -1.3032 | 2.4031 | 1.5362 |
| BCL2 | NM_000633 | -1.5431 | -2.4985 | -1.1355 | -2.5812 |
| BMP7 | NM_001719 | 3.025 | 6.8458 | 15.3499 | 10.4246 |
| CAV1 | NM_001753 | -1.082 | -1.5229 | -1.4654 | 1.166 |
| CCL11 | NM_002986 | 1.1886 | 1.8795 | 5.5835 | 6.1582 |
| CCL2 | NM_002982 | -1.2167 | 1.1992 | -1.1852 | -1.2448 |
| CCL3 | NM_002983 | -1.7001 | -1.037 | 1.8967 | -1.4953 |
| CCR2 | NM_001123396 | 1.3775 | 1.0767 | 4.4494 | 1.4762 |
| CEBPB | NM_005194 | -1.4934 | -1.3189 | 1.0914 | -1.1088 |
| COL1A2 | NM_000089 | -1.8643 | -1.7024 | 1.6104 | -1.9408 |
| COL3A1 | NM_000090 | 1.0928 | -1.1512 | 1.2528 | -2.8426 |
| CTGF | NM_001901 | -1.5521 | -2.2927 | -2.1585 | -1.6202 |
| CXCR4 | NM_003467 | -1.1056 | -4.2188 | -39.4729 | -24.4934 |
| DCN | NM_001920 | 1.3527 | 1.2393 | 1.6092 | -1.0201 |
| EDN1 | NM_001955 | 1.2781 | 2.5761 | 4.6958 | 1.8661 |
| EGF | NM_001963 | 1.0446 | 1.8147 | -1.0699 | 1.7418 |
| ENG | NM_000118 | -1.505 | -1.4637 | 1.0061 | -1.6834 |
| FASLG | NM_000639 | -1.0778 | 1.0767 | 26.0558 | 2.3434 |
| GREM1 | NM_013372 | -1.6359 | -1.331 | -33.5892 | 1.7391 |
| HGF | NM_000601 | -1.0195 | -1.6801 | -1.9191 | -1.6018 |
| IFNG | NM_000619 | -1.0778 | 1.0767 | -1.3034 | 1.3649 |
| IL10 | NM_000572 | -1.5479 | -1.4417 | 1.2579 | 1.3367 |
| IL13 | NM_002188 | 1.3767 | -1.1128 | 3.3541 | 1.2105 |
| IL13RA2 | NM_000640 | 1.0503 | -1.1115 | -1.4613 | -1.0277 |
| IL1A | NM_000575 | -1.1984 | 1.4435 | 2.5961 | 7.0587 |
| IL1B | NM_000576 | 1.0042 | -1.1513 | 4.8768 | 1.8792 |
| IL4 | NM_000589 | -1.0778 | 1.0767 | -1.3034 | 1.4298 |
| IL5 | NM_000879 | 1.0667 | 1.0826 | 2.8693 | 1.5514 |
| ILK | NM_004517 | -1.038 | 1.1531 | 1.4271 | -1.0824 |
| INHBE | NM_031479 | -1.3818 | -1.6081 | 4.9498 | 1.311 |
| ITGA1 | NM_181501 | -1.0437 | -1.0905 | -2.6436 | -1.0929 |
| ITGA2 | NM_002203 | -1.0912 | -1.738 | 3.0759 | 2.6884 |
| ITGA3 | NM_002204 | -1.0826 | -1.6058 | -1.4529 | 1.0635 |
| ITGAV | NM_002210 | -1.0675 | -1.4369 | 2.4538 | 1.5992 |
| ITGB1 | NM_002211 | -1.3462 | -1.2405 | 1.0291 | 1.2068 |
| ITGB3 | NM_000212 | -1.4745 | -1.6343 | -1.6563 | 1.0207 |
| ITGB5 | NM_002213 | -1.6439 | -2.5056 | -1.0154 | -1.0458 |
| ITGB6 | NM_000888 | 1.4291 | 1.8303 | 1.1062 | 2.3099 |
| ITGB8 | NM_002214 | -1.2327 | -1.4947 | 5.8549 | -2.8037 |
| JUN | NM_002228 | -1.0767 | -1.1654 | 1.0256 | 1.9381 |
| LOX | NM_002317 | 1.1912 | 1.1136 | 5.3666 | 4.0442 |
| LTBP1 | NM_000627 | -1.4974 | -1.7198 | 4.5213 | -1.1955 |
| MMP1 | NM_002421 | -1.1657 | -1.158 | -8.1134 | 1.4186 |
| MMP13 | NM_002427 | -1.0778 | 1.0767 | -1.3034 | 1.4008 |
| MMP14 | NM_004995 | -1.2915 | -1.3363 | 1.6815 | -1.7269 |
| MMP2 | NM_004530 | -1.3138 | -1.7309 | 1.42 | -1.3428 |
| MMP3 | NM_002422 | 1.279 | 1.0575 | -12.5001 | 7.2097 |
| MMP8 | NM_002424 | 1.1069 | 1.0767 | -1.1765 | 1.3981 |
| MMP9 | NM_004994 | 1.0833 | 2.2983 | 1.8757 | 7.4185 |
| MYC | NM_002467 | -1.1999 | -1.4481 | -1.6544 | -1.1563 |
| NFKB1 | NM_003998 | -1.1665 | -1.6345 | 1.5027 | 1.2309 |
| PDGFA | NM_002607 | -1.4048 | 1.1771 | -1.731 | 3.4891 |
| PDGFB | NM_002608 | 2.6837 | 4.6012 | 3.6691 | 7.1192 |
| PLAT | NM_000930 | -1.2926 | 1.1801 | 5.472 | 1.7465 |
| PLAU | NM_002658 | -1.4483 | -1.3753 | -1.0065 | -1.7144 |
| PLG | NM_000301 | -1.0778 | 1.0767 | -1.3034 | 1.4512 |
| SERPINA1 | NM_000295 | 2.8775 | 1.8623 | 7.5453 | 4.331 |
| SERPINE1 | NM_000602 | -1.2555 | -1.4394 | 1.0944 | 1.788 |
| SERPINH1 | NM_001235 | -1.5957 | -1.6339 | -1.4408 | -2.6914 |
| SMAD2 | NM_005901 | -1.464 | -2.305 | 1.0074 | -1.5516 |
| SMAD3 | NM_005902 | -1.3407 | -1.3129 | 2.0175 | -1.0082 |
| SMAD4 | NM_005359 | -1.551 | -1.4307 | 1.3594 | 1.1642 |
| SMAD6 | NM_005585 | -1.2144 | -1.7115 | 5.0986 | 1.9831 |
| SMAD7 | NM_005904 | -1.2416 | 2.3501 | 2.8963 | 2.839 |
| SNAI1 | NM_005985 | -1.0886 | 6.4751 | 3.9061 | 4.9082 |
| SP1 | NM_138473 | 1.1241 | -1.2684 | 1.6479 | 1.0779 |
| STAT1 | NM_007315 | -1.836 | -1.9628 | -1.2228 | -2.0745 |
| STAT6 | NM_003153 | -1.0511 | -1.3289 | 2.0607 | -1.1864 |
| TGFB1 | NM_000660 | -1.2857 | -1.5306 | 1.7761 | 1.4796 |
| TGFB2 | NM_003238 | -1.251 | -1.0256 | 2.2636 | -1.4564 |
| TGFB3 | NM_003239 | -1.5771 | -1.0504 | -2.0662 | -7.025 |
| TGFBR1 | NM_004612 | -1.7125 | -1.033 | 1.126 | -1.8018 |
| TGFBR2 | NM_003242 | -1.5856 | -2.1114 | -1.0377 | -1.7238 |
| TGIF1 | NM_003244 | -1.3719 | -1.0115 | -1.3908 | -2.4521 |
| TNF | NM_000594 | -1.1203 | 1.0533 | -2.2337 | 25.5612 |
| VEGFA | NM_003376 | 1.1214 | 1.0487 | 3.6872 | 3.9302 |

**Supplementary Table1**. **Fibrotic gene transcript profile in PHCM exposed to *T. cruzi*.** Total RNA purified from serum starved PHCM monolayers exposed to invasive *T. cruzi* trypomastigotes used for the microarray assay were also used in a fibrotic PCR array assay to evaluate gene transcript profiles by qPCR. Total RNA (500ng) purified from the cells were converted to cDNA and used for antifibrotic gene transcript quantitation by fibrosis RT² Profiler PCR Arrays. The relative expression of each transcript was normalized against housekeeping genes. Each value is the mean of biological triplicates performed in technical duplicates. The p value for each fold change in the table is less than 0.05.
